# RSL3 Drives Ferroptosis Through GPX4 Inactivation and ROS Production in Colorectal Cancer

**DOI:** 10.3389/fphar.2018.01371

**Published:** 2018-11-22

**Authors:** Xinbing Sui, Ruonan Zhang, Shuiping Liu, Ting Duan, Lijuan Zhai, Mingming Zhang, Xuemeng Han, Yu Xiang, Xingxing Huang, Haoming Lin, Tian Xie

**Affiliations:** ^1^Department of Medical Oncology, Holistic Integrative Oncology Institutes and Holistic Integrative Cancer Center of Traditional Chinese and Western Medicine, The Affiliated Hospital of Hangzhou Normal University, College of Medicine, Hangzhou Normal University, Hangzhou, China; ^2^Department of Cancer Pharmacology, Holistic Integrative Pharmacy Institutes, College of Medicine, Hangzhou Normal University, Hangzhou, China; ^3^Key Laboratory of Elemene Class Anti-cancer Chinese Medicine of Zhejiang Province and Engineering Laboratory of Development and Application of Traditional Chinese Medicine from Zhejiang Province, Hangzhou Normal University, Hangzhou, China; ^4^Department of Hepatobiliary Pancreatic Surgery, Sun Yat-sen Memorial Hospital, Sun Yat-sen University, Guangzhou, China

**Keywords:** RSL3, ferroptosis, glutathione peroxidase 4, reactive oxygen species, colorectal cancer

## Abstract

Ferroptosis is an iron-dependent, oxidative cell death, and is characterized by iron-dependent accumulation of reactive oxygen species (ROS) within the cell. It has been implicated in various human diseases, including cancer. Recently, ferroptosis, as a non-apoptotic form of cell death, is emerging in specific cancer types; however, its relevance in colorectal cancer (CRC) is unexplored and remains unclear. Here, we showed that ferroptosis inducer RSL3 initiated cell death and ROS accumulation in HCT116, LoVo, and HT29 CRC cells over a 24 h time course. Furthermore, we found that ROS levels and transferrin expression were elevated in CRC cells treated with RSL3 accompanied by a decrease in the expression of glutathione peroxidase 4 (GPX4), indicating an iron-dependent cell death, ferroptosis. Overexpression GPX4 resulted in decreased cell death after RSL3 treatment. Therefore, RSL3 was able to induce ferroptosis on three different CRC cell lines *in vitro* in a dose- and time-dependent manner, which was due to increased ROS and an increase in the cellular labile iron pool. Moreover, this effect was able to be reversed by overexpression of GPX4. Taken together, our results suggest that the induction of ferroptosis contributed to RSL3-induced cell death in CRC cells and ferroptosis may be a pervasive and dynamic form of cell death for cancer treatment.

## Introduction

Ferroptosis emerging as a new programmed cell death is form, which is genetically, biochemically, and morphologically distinct from necrosis, autophagy, and apoptosis (Yang and Stockwell, 2016). This process is characterized by suppression of the phospholipid glutathione peroxidase 4 (GPX4) and subsequent intracellular accumulation of lipid reactive oxygen species (ROS) in an iron-dependent manner (Dixon et al., 2012; Yang et al., 2014). Ferroptosis can be induced by some small molecules, including erastin and RSL3. Erastin can activate ferroptosis by directly suppressing the cell surface cysteine-glutamate antiporter (system X_c_^-^), resulting in the inactivation of the cellular antioxidant glutathione (GSH) (Dixon et al., 2014; Morris et al., 2018). Whereas, RSL3 is identified as a potent ferroptosis-triggering agent, which is dependent on the activity of GPX4 (Shintoku et al., 2017). GPX4, as a negative target of RSL3, is demonstrated to mediate suppression of ferroptosis (Friedmann Angeli et al., 2014; Yang et al., 2014). In addition, several genes or proteins have been also involved in the process of ferroptosis. Ras transformation is demonstrated to render cells sensitive to ROS-induced ferroptosis; however, in some cases RAS-driven nuclear factor erythroid 2-related factor 2 (NRF2) activation can enhance the expression of antioxidant genes that protects hepatocellular carcinoma (HCC) cells against ferroptosis (Schott et al., 2015; Sun et al., 2016). Meanwhile, p53 contributes to ROS-induced ferroptosis by transcriptional repression of solute carrier family 7 member 11 (SLC7A11) (Jiang et al., 2015). Although a number of key targets has been demonstrated to be the important mediators that induce ferroptosis, it still unclear whether these targets are susceptible to ferroptosis-induced cell death.

In cancer cells, erastin or RSL3 can promote ferroptosis-induced cell death. However, ferroptosis has not been investigated directly in cancer therapy. Therefore, exploitation of ferroptosis for killing cancer cells in response to specific compounds are attracting more and more attention in cancer therapy. We investigated the effect of RSL3 (a selective ferroptosis inducer) on the viability of three different CRC cells and found RSL3 triggered ferroptotic cell death in a dose- and time-dependent manner, which was due to increased ROS levels and an increase in the cellular labile iron pool. In further analysis, we demonstrated that GPX4 inhibition was a key determinant in RSL3-induced ferroptosis. Overexpression GPX4 rescued the ferroptotic cell death induced by RSL3 treatment. Our results provide a promising strategy to for the treatment of colorectal cancer (CRC) patients.

## Materials and Methods

### Cell Culture and Reagents

Human CRC cell lines (HCT116, LoVo, HT29) were purchased from ATCC, these cells were cultured in McCoy’s 5A or Dulbecco’s modified Eagle’s medium (DMEM; Gibco BRL, Rockville, MD, United States) with 10% heat-inactivated fetal bovine serum at 37°C, 95% humidity, and 5% CO_2_. The fetal bovine serum was purchased from Corille (184590, Australia). RSL3 (#S8155) and liproxstatin-1(#S7699) and ferrostatin-1 (#S7243) were purchased from Selleck Chemical (Houston, TX, United States). Z-VAD-FMK (#V116), deferoxamine (#D9533), necrostatin-1 (#N9037), chloroquine (#C6628), and doxorubicin (#D1515) were obtained from Sigma. Ferritin (ab75973), transferrin (ab82411) and GPX4 (ab125066) were purchased form Abcam; GAPDH (#5174) was obtained from Cell Signaling Technology (CST). GPX4 plasmid (RG208065) was purchased from OriGene.

### Cell Viability Assay

Cell viability was evaluated using the Cell Counting Kit-8 (CCK-8) (LJ621, Dojindo, Japan) according to the manufacturer’s instructions. Cells were seeded in 96-well flat bottom microtiter plates at a density of 5,000 cells per well. Twenty-four hours later, RSL3 was added at the concentrations indicated for 6, 12, 24, and 48 h. The absorbance was measured on a microplate reader (Synergy HT, Bio-Tek, United States) at 450 nm.

### Determination of Cell Death

Pharmingen annexin V-FITC Apoptosis Detection Kit I (BD, United States) was used to detect apoptosis and the estimation procedure was performed according to the manufacturer’s instructions.10^6^ cells were seeded into a 6 cm dish. After attachment overnight, cells were washed twice with PBS and the medium was replaced medium with 3 μM RSL3 (or combination with 1 μM liproxstatin-1) for 24 h. All cells including the floating cells in the culture medium were harvested. Cell were washed, trypsinized, and pelletized together with the supernatant by centrifugation. After repeated washing, the cells were resuspended in ice-cold 1× binding buffer at a concentration of 1 × 10^6^ cells/ml. 100 μl of cell suspension were each mixed with 5 μl FITC annexin V and 5 μl PI. The mixture was incubated for 15 min at room temperature in the dark and then analyzed by FACS Calibur Flow Cytometer (Beckman Coulter, CytoFLEX S, United States).

### Measurement of the Labile Iron Pool (LIP)

Labile iron pool (LIP) was measured according to the methods described by the manufacturer’s instructions (Prus and Fibach, 2008). Briefly, cells were trypsinized, washed twice with 0.5 ml of PBS, and incubated at a density of 1 × 10^6^/ml for 15 min at 37°C with 0.05 μM calcein-acetoxymethyl ester (AnaSpec). Then, the cells were washed twice with 0.5 ml of PBS and either incubated with deferiprone (100 μM) for 1 h at 37°C or left untreated. The cells were analyzed with a flow cytometer. Calcein was excited at 488 nm, and fluorescence was measured at 525 nm. The difference in the cellular mean fluorescence with and without deferiprone incubation reflects the amount of LIP.

### Determination of ROS Generation

Change in intracellular ROS levels were by measuring the oxidative conversion of cell permeable 2′,7′-dichlorofluorescein diacetate (DCFH-DA, Sigma) to fluorospectro-photometer (Chen et al., 2018). Cells were plated in 6 cm dish (1 × 10^6^ cells/well) and allowed to attach overnight. Cells were incubated with control media or RSL3 for 24 h. The cells were washed with D-Hank’s and incubated with DCFH-DA at 37°C for 20 min. Then DCF fluorescence distribution of 20,000 cells was detected by FACS Calibur Flow Cytometer (Beckman Coulter, CytoFLEX S, United States) at an excitation wavelength of 488 nm and at an emission wavelength of 535 nm.

### GPX4-Expressing Plasmid and Transfection

Human CRC cell lines (HCT116, LoVo, HT29) were transiently transfected with GPX4-expressing plasmid or empty vector for 36 h, using MegaTran 1.0 transfection reagent (OriGene). Then, cells were harvested for further study.

### Western Blot Assay

Cells were harvested from cultured dishes and were lysed in a lysis buffer [20 mM Tris-HCl pH 7.6, 1 mM EDTA, 140 mM NaCl, 1% NP-40, 1% aprotinin, 1 mM phenylmethylsulfonyl fluoride (PMSF), 1 mM sodium vanadate]. Protein concentration was determined using a BCA Protein Assay Kit (P0009, Beyotime). Cell lysates (40 mg protein/line) were separated on a 5–20% Tris-Tricine Ready Gel SDSPAGE (Bio-Rad) for nitrocellulose membrane blotting. The blotted membranes were blocked with 5% skim milk for 1 h and were incubated with primary antibodies. The immunoreactive bands were visualized by enhanced chemiluminescence using horseradish perox-idase-conjugated IgG secondary antibodies. Band density was measured by densitometry, quantified using gel plotting macros of NIH image 1.62, and normalized to an indicated sample in the identical membrane.

### Statistical Analysis

Data are expressed as means ± SD of three independent experiments. Statistical analysis was performed using Prism 7.0c GraphPad Software. The significance of differences between groups was determined using *t*-test. A *p*-value < 0.05 was considered statistically significant.

## Results and Discussion

### RSL3 Trigger Cell Death in Colorectal Cancer Cells

**FIGURE 1:**
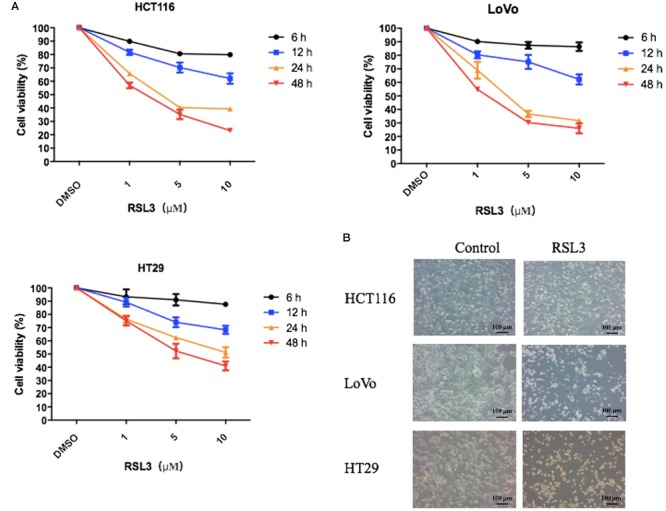
RSL3 selectively induces growth inhibition in CRC cells in concentration- and time-dependent manner. **(A)** The cell viability was measured using the Cell Counting Kit-8 (CCK-8). RSL3 was dissolved in DMSO and the cell viability of DMSO was considered as 100%. The experiments were performed in triplicate. **(B)** Representative cell morphological changes are detected by light microscopy.

RSL3 (RAS-selective lethal) is a ferroptosis-inducing agent. The previous reports showed RSL3 could kill RAS-transformed tumorigenic fibroblast cell lines and irreversibly inactivate GPX4. To identify whether RSL3 induces growth suppression in CRC cells, three human colorectal cell lines were treated with various concentrations of RSL3 at different times and the cell viability was assessed by the Cell Counting Kit-8 (CCK-8). We found RSL induced cell death in CRC cells in dose- and time-dependent manner (Figure 1). The 24 h IC50 of RSL3 for HCT116, LoVo, and HT29 was 4.084 μM, 2.75 μM, and 12.38 μM, respectively. Consequently, we used 3 μM RSL3 in HCT116, LoVo, and HT29 cells for 24 h in subsequent experiments. Interestingly, RSL3 induced growth inhibition not only in K-ras mutant HCT116 and LoVo cells but also in K-ras wild type HT29 cell. Therefore, RAS mutation status appeared to have no impact on the cell viability of CRC cells after RSL3 treatment. Taken together, our results suggest that RSL3 selectively induces growth inhibition in CRC cells in dose- and time-dependent manner but not RAS-dependent manner.

### Ferroptosis Contributes to RSL3-Induced Growth Inhibition in CRC Cells

**FIGURE 2:**
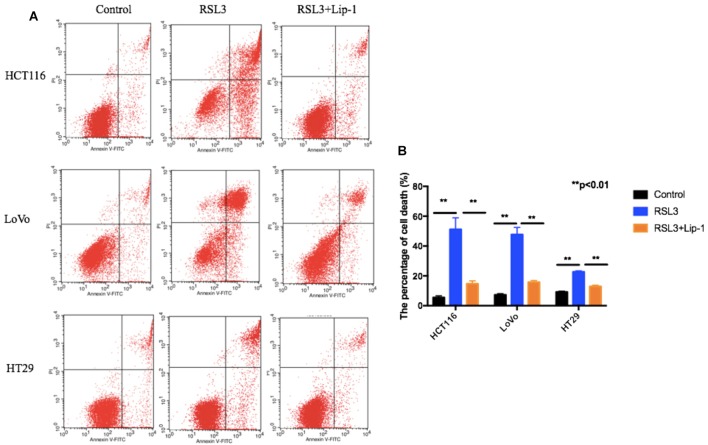
Representative results of annexin V-FITC/PI staining **(A)** and quantitative analysis **(B)**; values are mean ± SD of three independent experiments; ^∗∗^*p* < 0.01.

To assess whether ferroptosis contributed to the growth inhibition to RSL3 in CRC cells, we treated these cells with liproxstatin-1 (Lip-1). Lip-1, a potent pharmacological inhibitor of ferroptosis, is demonstrated to suppress ferroptosis via lipid peroxide scavenging in an inducible mouse model of GPX4 depletion as well as in immortalized fibroblasts in the absence of GPX4 (Kim et al., 2016). Then, cell death was assessed by flow cytometry. As shown in Figures 2A,B, three CRC cells displayed extensive percentage of dead cells after RSL3 and pre-treatment of these cells with the ferroptosis inhibitor Lip-1 rescued RSL3-induced cell death.

**FIGURE 3:**
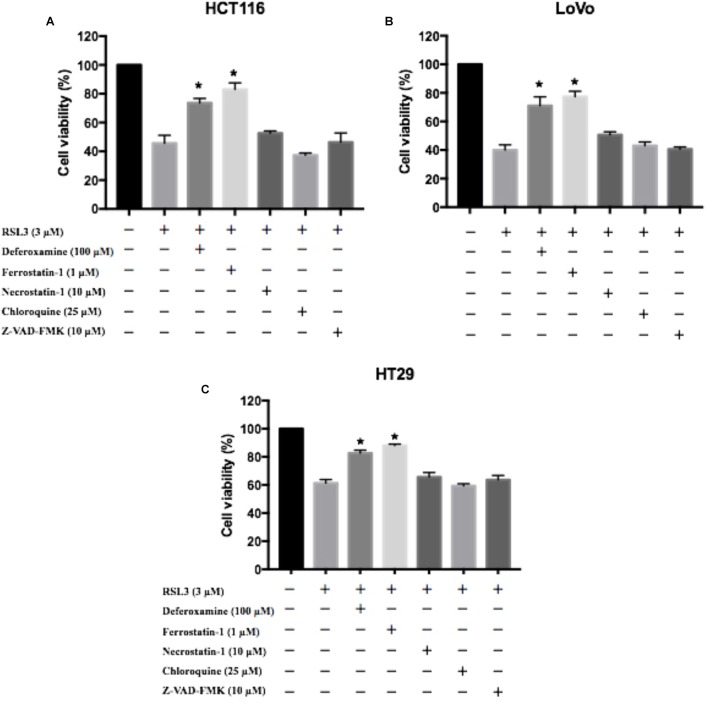
Ferroptosis contributes to RSL3-induced growth inhibition in CRC cells. **(A)** HCT116 cells were treated with RSL3 with or without the indicated inhibitors for 24 h and cell viability was assayed (*n* = 3, ^∗^*p* < 0.05 *versus* RSL3 treatment group); **(B)** HT29 cells were treated with RSL3 with or without the indicated inhibitors for 24 h and cell viability was assayed (*n* = 3, ^∗^*p* < 0.05 *versus* RSL3 treatment group). **(C)** LoVo cells were treated with RSL3 with or without the indicated inhibitors for 24 h and cell viability was assayed (*n* = 3, ^∗^*p* < 0.05 *versus* RSL3 treatment group).

To further determine the role of in RSL3-induced cell death, HCT116, LoVo, and HT29 cells were treated with RSL3 in the absence or presence of several cell death inhibitors. The treatment combined with deferoxamine (an iron-chelating agent), ferrostatin-1 (a potent inhibitor of ferroptosis), but not with necrostatin-1 (a potent inhibitor of necroptosis), chloroquine (a potent inhibitor of autophagy) or Z-VAD-FMK (a general caspase inhibitor), prevented RSL3-induced growth inhibition in these cells (Figure 3). Thus, these data indicate that ferroptosis may contributes to RSL3-induced growth inhibition in CRC cells.

### RLS3 Promotes Ferroptosis-Associated LIP Increase and ROS Accumulation

**FIGURE 4:**
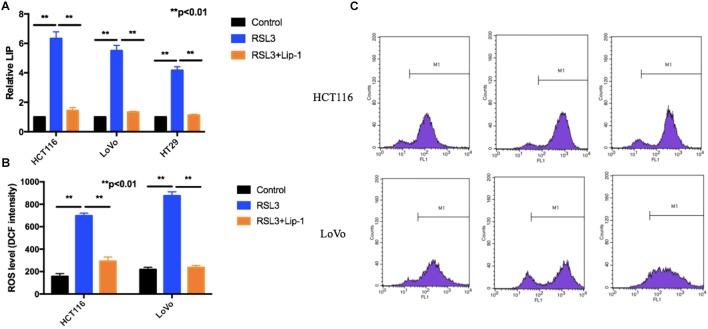
RSL3 promotes ferroptosis-associated LIP increase and ROS accumulation. **(A)** The cellular LIP was analyzed with a flow cytometer. **(B)** Representative results of using an oxidation-sensitive fluorescent probe, DCFH-DA. **(C)** Values are mean ± SD of three independent experiments; ^∗∗^*p* < 0.01.

Iron is the essential reactive element for many biological processes including ROS generation reaction and ferroptosis. Moreover, the LIP, as the crossroad of cellular iron traffic, was reported to be associated with ferroptosis by directly catalyzing ROS generation (Prus and Fibach, 2008; Doll and Conrad, 2017). We first detected the cellular LIP. RSL3 treatment triggered an increase of the cellular LIP (Figure 4A). Then, we measured if suppression of ferroptosis can block RSL3-induced LIP increase. As shown in Figure 4A, Lip-1 can block ferroptosis-associated LIP increase.

Reactive oxygen species accumulation is regarded as one hallmark of ferroptosis. Increasing data show that various ROS scavengers and ferroptosis inhibitors can entirely repress ferroptotic cell death and cellular ROS accumulation (Conrad et al., 2018; Sun et al., 2018). To determine whether ROS played a key role in RSL3-induced cell death, we measured intracellular ROS levels by using an oxidation-sensitive fluorescent probe DCFH-DA, which is oxidized to DCF in the presence of ROS. Our results showed RSL-3 increased intracellular ROS levels which was represented by the DCF intensity (Figures 4B,C). Moreover, this effect can be rescued by the treatment with Lip-1 (Figures 4B,C). In summary, these data suggest RSL3 promotes ferroptosis-associated LIP increase and ROS accumulation.

### GPX4 Suppression and Transferrin Activation Contribute to RSL-3 Induced Ferroptosis

**FIGURE 5:**
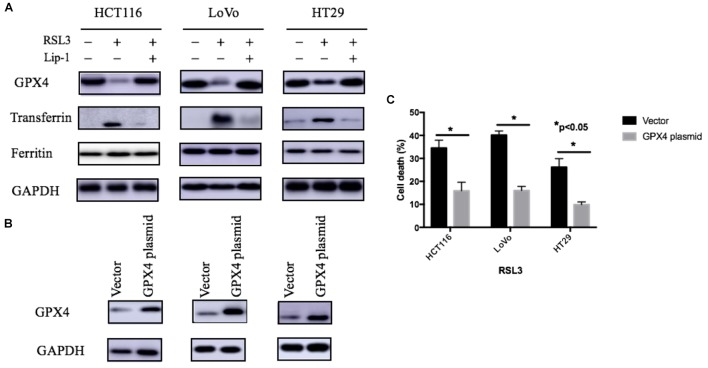
Expression of iron regulatory proteins **(A)** and the effect of GPX4 on RSL-3-induced cell death through ferroptosis **(B)**. **(C)** Values are mean ± SD of three independent experiments; ^∗∗^*p* < 0.05.

GPX4 is one of the most important antioxidant enzymes and an essential regulator of ferroptotic cancer cell death. The current studies have showed that the activation of GPX4 can suppress ferroptosis and inflammation (Wenzel et al., 2017; Ingold et al., 2018). As agreement with these reports, our study showed RSL-3 could inhibit GPX4 expression and this may subsequently have a critical role in RSL-3-induced ferroptosis (Figure 5A). Moreover, overexpression GPX4 resulted in decreased cell death after RSL3 treatment (Figures 5B,C). Therefore, GPX4 inhibition could be a key determinant in RSL3-induced ferroptosis.

As we know, the ROS generation LIP rise could be attributed to either the degradation of cellular iron storage protein ferritin or the accumulation of iron uptake. Transferrin-mediated iron transport into cells is the most common iron uptake pathway, and transferrin is a key regulator for ferroptosis (Gkouvatsos et al., 2012; Ward and Kaplan, 2012; Gao et al., 2015). To investigate whether iron regulatory proteins such as ferritin and transferrin are changed after RSL-3 treatment, we performed Western blotting analysis to determine the expression of iron regulatory proteins. The expression of transferrin significantly increased after RSL-3 treatment (Figure 5A). To make sure this effect was not attributed to the alteration in iron transport regulatory proteins, we detected the expression of transferrin receptor ferritin and found that transferrin was not significantly changes after RSL-3 treatment (Figure 5A). Our results suggest RSL3 treatment triggers ferroptotic cell death in CRC cells through altering iron transport.

## Conclusion

Ferroptosis is dependent upon intracellular iron, and is a new cell death form distinct from necrosis, autophagy, and apoptosis (Dar et al., 2018; Gao et al., 2018). Ferroptosis is tightly controlled by GPX4 and some iron transport regulatory proteins (Feng and Stockwell, 2018). Exploitation of ferroptosis for killing cancer cells in response to specific compounds are attracting more and more attention in cancer therapy. The ferroptosis trigger erastin can overcome the drug resistance of chemotherapeutic agents in acute myeloid leukemia cells (Yu et al., 2015). Sorafenib is shown to induce ferroptosis in iron-dependent oxidative mechanisms, which open new avenues for the optimization of clinical application of sorafenib in the treatment for HCC (Louandre et al., 2013). Artesunate (ART), an anti-malarial, induces an iron- and ROS-dependent cell killing in pancreatic ductal adenocarcinoma cell lines with wild-type and mutant K-Ras, which indicates ART can function as a specific inducer of ferroptosis in pancreatic cancer cells (Eling et al., 2015). However, the role of ferroptosis in CRC is still unclear.

In this study, we for the first time reported RSL3 triggered ferroptotic cell death by promoting the accumulation of cellular ROS and increasing the cellular LIP level. Mechanismly, we found transferrin expression were elevated in CRC cells treated with RSL3 accompanied by a decrease in the expression of GPX4, indicating an iron-dependent cell death. Moreover, overexpression GPX4 rescued the ferroptotic cell death induced by RSL3 treatment, indicating that GPX4 inhibition could be a key determinant in RSL3-induced ferroptosis in CRC cells. Our results may provide a pervasive and dynamic form of cell death for CRC treatment, however, this study is lack of sufficient experimental evidence to support our conclusions and this research is based on *in vitro* findings, which do not always extrapolate to the *in vivo* setting. In the future, we will make further investigations for this limitations.

## Author Contributions

XS, TX, and HL conceived the idea and designed the study. RZ and XS performed all the experiments, analyzed the data, and co-wrote the paper. TD, LZ, MZ, XmH, YX, and XxH provided technical support. SL helped correcting the manuscript. All the authors read and approved the final version of the manuscript prior to submission.

## Conflict of Interest Statement

The authors declare that the research was conducted in the absence of any commercial or financial relationships that could be construed as a potential conflict of interest.
